# Clinical scores and blood biomarkers for Prediction of bacteremia in emergency department patients: Bacteremia Assessment in Clinical Triage (BACT) study

**DOI:** 10.1186/cc14088

**Published:** 2015-03-16

**Authors:** S Laukemann, N Kasper, N Kasper, A Kutz, S Felder, S Haubitz, B Müller, P Schuetz

**Affiliations:** 1 Kantonsspital Aarau, Switzerland; 2 University Hospital, Bern, Switzerland

## Introduction

Collection of blood cultures is routinely performed in patients with suspicion of infection in the emergency department (ED) despite a low yield of positive culture results. To increase sensitivity, different clinical prediction rules and blood biomarkers have been put forward. Herein, we validated the performance of different promising clinical prediction rules alone and in combination with novel blood biomarkers to predict blood culture positivity.

## Methods

This is an observational cohort study including consecutive medical patients with suspected infection and collection of ED admission blood cultures. Five clinical prediction rules were calculated and admission concentrations of procalcitonin (PCT), C-reactive protein, neutrophil-lymphocyte count ratio (NLCR), lymphocyte count, white blood cell count, and red blood cell distribution width were measured. True blood culture positivity was assessed by two independent physicians. We used logistic regression models with area under the curve (AUC) to establish associations between clinical prediction rules and blood culture positivity.

## Results

Of 1,083 included patients, 106 (9.8%) cultures were positive. Of the clinical prediction rules, the Shapiro rule performed best (AUC 0.733) followed by the Metersky rule (AUC 0.609). The best biomarkers were PCT (AUC 0.796), NLCR (0.692) and lymphocyte count (AUC 0.671). Combination of the Shapiro rule and PCT showed the best combination result (AUC of combined model 0.822). Limiting blood cultures to either the Shapiro rule ≥4 points or PCT >0.11 μg/l would reduce negative sampling by 25.6% while still identifying 100% of positive cultures. Using a Shapiro rule ≥3 points or PCT >0.25 μg/l limit would reduce negative sampling by 42.1% while still identifying 96.2% of positive cultures.

## Conclusion

Combination of clinical parameters combined in the Shapiro rule together with admission levels of PCT allows reduction of unnecessary blood cultures with minimal false negative rates.
